# Box-Behnken Design-Based Optimization and Evaluation of Lipid-Based Nano Drug Delivery System for Brain Targeting of Bromocriptine

**DOI:** 10.3390/ph17060720

**Published:** 2024-06-02

**Authors:** Asha Spandana K M, Mohit Angolkar, Mohamed Rahamathulla, Kamal Y. Thajudeen, Mohammed Muqtader Ahmed, Syeda Ayesha Farhana, Thippeswamy Boreddy Shivanandappa, Sharanya Paramshetti, Riyaz Ali M. Osmani, Jawahar Natarajan

**Affiliations:** 1 Department of Pharmaceutics, JSS College of Pharmacy-Mysuru, JSS Academy of Higher Education and Research, Mysuru 570015, India; asha@jssuni.edu.in (A.S.K.M.); mohitangolkar11@gmail.com (M.A.); paramshettisharanya@gmail.com (S.P.); riyazosmani@gmail.com (R.A.M.O.); 2 Department of Pharmaceutics, College of Pharmacy, King Khalid University, Al Faraa, Abha 62223, Saudi Arabia; rahapharm@gmail.com; 3 Department of Pharmacognosy, College of Pharmacy, King Khalid University, Al Faraa, Abha 62223, Saudi Arabia; kthajudeen@kku.edu.sa; 4 Department of Pharmaceutics, College of Pharmacy, Prince Sattam Bin Abdul Aziz University, Al Kharj 11942, Saudi Arabia; muqtadernano@psau.edu.sa; 5 Department of Pharmaceutics, College of Pharmacy, Qassim University, Buraidah 51452, Saudi Arabia; a.farhana@qu.edu.sa; 6 Department of Biomedical Science, College of Pharmacy, Shaqra University, Al-Dawadmi Campus, Al-Dawadmi 11961, Saudi Arabia; drswamy@su.edu.sa; 7 Department of Pharmaceutics, JSS College of Pharmacy-Ootacamund, JSS Academy of Higher Education and Research, Mysuru 570015, India

**Keywords:** blood–brain barrier, solid lipid nanoparticles, bromocriptine, nanostructured lipid carriers, Parkinson’s disease

## Abstract

Bromocriptine (BCR) presents poor bioavailability when administered orally because of its low solubility and prolonged first-pass metabolism. This poses a significant challenge in its utilization as an effective treatment for managing Parkinson’s disease (PD). The utilization of lipid nanoparticles can be a promising approach to overcome the limitations of BCR bioavailability. The aim of the research work was to develop and evaluate bromocriptine-loaded solid lipid nanoparticles (BCR-SLN) and bromocriptine-loaded nanostructured lipid carriers (BCR-NLC) employing the Box-Behnken design (BBD). BCR-SLNs and BCR-NLCs were developed using the high-pressure homogenization method. The prepared nanoparticles were characterized for particle size (PS), polydispersity index (PDI), and entrapment efficiency (EE). In vitro drug release, cytotoxicity studies, in vivo plasma pharmacokinetic, and brain distribution studies evaluated the optimized lipid nanoparticles. The optimized BCR-SLN had a PS of 219.21 ± 1.3 nm, PDI of 0.22 ± 0.02, and EE of 72.2 ± 0.5. The PS, PDI, and EE of optimized BCR-NLC formulation were found to be 182.87 ± 2.2, 0.16 ± 0.004, and 83.57 ± 1.8, respectively. The in vitro release profile of BCR-SLN and BCR-NLC showed a biphasic pattern, immediate release, and then trailed due to the sustained release. Furthermore, a pharmacokinetic study indicated that both the optimized BCR-SLN and BCR-NLC formulations improve the plasma and brain bioavailability of the drug compared to the BCR solution. Based on the research findings, it can be concluded that the BCR-loaded lipid nanoparticles could be a promising carrier by enhancing the BBB penetration of the drug and helping in the improvement of the bioavailability and therapeutic efficacy of BCR in the management of PD.

## 1. Introduction

The neurodegenerative disorder, such as Parkinson’s disease (PD), is a condition characterized pathologically by dopaminergic neuron loss in the nigrostriatal area [1]. Parkinson’s disease (PD) especially affects the mesencephalon, where slow neuronal degeneration takes place. The reasons are unknown, but genetic and toxic risk factors have been found. A prominent pathophysiological feature of Parkinson’s disease (PD) is the deterioration of the substantia nigra (SN), which results in the death of dopaminergic neurons and, specifically, disarray of the intricate circuits of the basal ganglia (BG). The synthesis of dopamine neurotransmitters is reduced as a consequence of damage to dopaminergic neurons, which may alter motor nerve activity. The goal of pharmacological therapy has been to restore dopaminergic neurotransmission. L-3,4-dihydroxyphenylalanine (L-DOPA), a dopamine precursor, is still the gold standard for PD therapy because it improves motor functions. On the other hand, L-DOPA also has many pharmacokinetic drawbacks [2], and there has been significant development in novel treatment methods for providing continuous dopaminergic stimulation (CDS). This treatment method aims to decrease the prevalence and severity of L-DOPA-related motor fluctuation and dyskinesia while also providing long-term safety and tolerance. CDS can be accomplished by administering a long-acting oral dopamine (DA) agonist [1].

The main limitation, however, is the design of a suitable carrier for the delivery of drugs to CNS. The BBB prevents most CNS drug candidates from penetrating the brain [3]. The BBB often obstructs the passage of intravenously (i.v.) delivered therapeutics into the cerebral compartment because it expresses efflux pumps, such as P-glycoprotein, which actively removes drugs from the brain [4,5]. However, most small particles (mw > 500 Daltons, D), proteins, and peptides do not permeate the BBB. Most chemicals must thus cross the BBB via interacting with particular transporters and/or receptors expressed on the luminal (blood) side of endothelial cells in order to reach the brain [6]. A potential approach to overcome these challenges includes the development of suitable novel drug carrier platforms. Recent advancements in nanotechnology have emerged as powerful tools for treating CNS disorders. Nanotechnology-based drug delivery systems provide remarkable opportunities to overcome the Blood-brain barrier (BBB) obstacle for the management of Central Nervous System (CNS) diseases. The brain is one of the susceptible and fragile neuronal organs present in the body. By regulating the intake of chemicals that are required (endogenous nutrients) and the outflow of toxic substances, the CNS barrier ensures brain homeostasis. The brain is mainly composed of BBB and blood-cerebrospinal fluid barrier (BCFB). By preventing the entry of CNS therapeutics, the BBB limits therapy options for CNS disorders. Nanoparticle drug delivery technologies provide significant benefits in terms of targeted drug delivery. The size of the particle determines the efficiency of the drug delivery system. The efficacy of nanoparticles for appropriate bioavailability and precise intracellular absorption of the active therapeutic moiety in the specified cellular targets is enhanced by their increased surface area and smaller size (nm) [7].

Currently, drugs that exhibit poor brain distribution can be loaded into nanoparticles to selectively bind to the receptors or transporters expressed at the BBB, resulting in improved CNS selectivity and permeability. Overall, these nanocarriers have resulted in better drug pharmacokinetics, effectiveness, and safety [8]. Considering the effectiveness of nanoparticles in crossing BBB and their limitations, particularly in terms of toxicity and stability, SLNs are another better solution for drug administration into the brain [9].

Dopamine (DA) receptor agonist BCR has been widely used in Parkinson’s disease clinical applications in order to delay and minimize the motor fluctuations driven by long-term levodopa treatment. It has been demonstrated that BCR has antioxidant properties, scavenges free radicals, inhibits the generation of free radicals, and suppresses apoptosis, which is thought to be the underlying cause of Parkinson’s disease. BCR is a substrate of CYP3A4, which is extensively metabolized in the gastrointestinal tract and liver by the CYP3A4 cytochrome system [10]. BCR is highly absorbed via the GI tract but has poor bioavailability due to the hepatic first-pass mechanism; nonetheless, several individuals have reported GI side effects such as headache and dizziness. To enhance the BCR delivery to the brain, i.v., vaginal, transdermal, and intranasal (IN) administration routes have been studied. A long-acting injectable formulation of BCR has been developed [11], which eliminates gastrointestinal side effects, but it is costly and cannot be self-administered. IV administration requires the use of co-solvents, which causes pain during injection and increases the risk of venous thrombosis [12], low patient compliance, and interindividual variability limits [13,14,15]. Previous studies have shown to improve the brain delivery of various therapeutic agents using SLN and NLC [16,17,18,19,20,21,22,23]. Nagaraj et al. developed SLN containing Zotepine to improve the oral bioavailability of the drug. Pharmacokinetics studies in Wistar rats exhibited a significantly higher AUC (1.3-fold) compared to Zotepine-coarse suspension [16]. Zhao B et al. prepared SLN containing hydroxysafflor yellow A (HSYA) for oral delivery, and HSYA nanoparticles showed a 3.97-fold increase in absorption compared to the HSYA solution [17]. Pramod Kumar et al. prepared methylthioadenosine (MTA) containing SLN for brain delivery; the pk study showed that SLN-encapsulated MTA could be effectively delivered to the brain and promote the remyelination of the neurons [18]. A study on Asenapine Maleate SLN by Vaishali M et al. revealed enhanced drug permeation through the BBB and increased accumulation within the brain [19]. Khan et al. developed NLC containing Atazanavir (ATZ) to improve brain availability. The prepared NLC showed biphasic release and an in vivo study demonstrated a 4-fold improvement in brain bioavailability of ATZ-NLC compared to the suspension using oral administration [20]. Eleraky et al. developed NLC containing Temazepam to improve brain availability. The prepared NLC showed sustained drug release, and the in vivo study demonstrated a significant increase in the bioavailability of the drug compared to the suspension using oral administration [12]. Therefore, the proposed research aims to develop and analyze solid lipid nanoparticles (SLNs) and nanostructured lipid carriers (NLCs) containing BCR for oral delivery with tailored physicochemical attributes to address the challenges of solubility and bioavailability associated with the drug BCR, classified as BCS Class II, in order to enhance its efficacy in treating Parkinson’s disease.

## 2. Result and Discussion

### 2.1. Screening of Lipids and Surfactant

**Figure 1:**
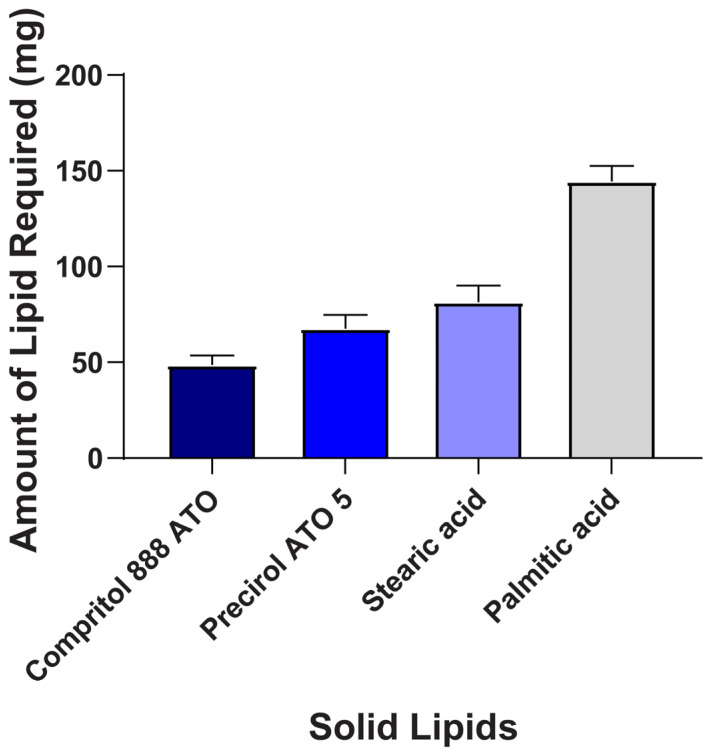
Solubility of BCR in solid lipids. Each value represents mean ± SD and the experiment was performed in a replicate of three (*n* = 3).

**Figure 2:**
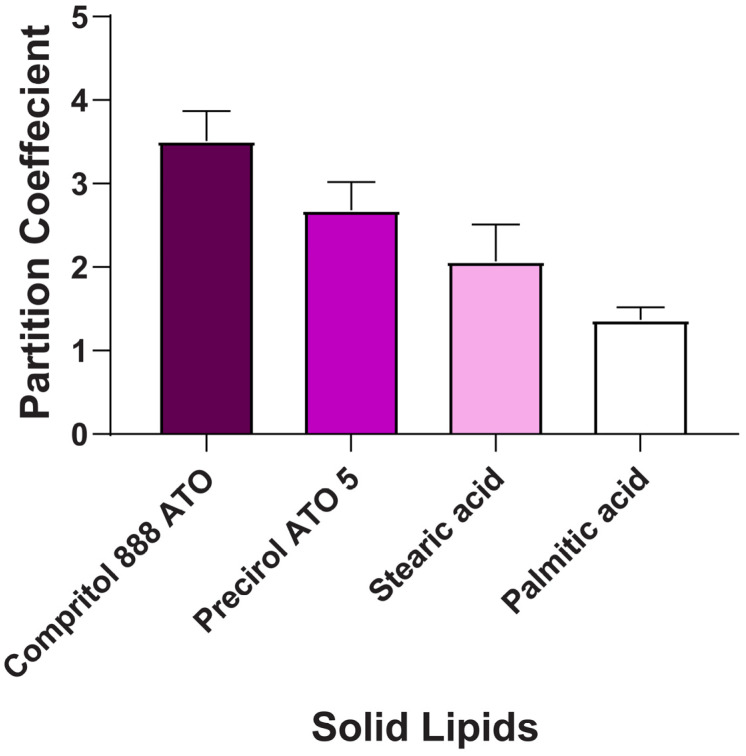
Partition Coefficient of BCR in solid lipids. Each value represents mean ± SD and the experiment was performed in a replicate of three (*n* = 3).

The results revealed that BCR had higher solubility and partitioning in Compritol 888 ATO (Lipid A) (3.45 ± 0.2) compared to other lipids, as depicted in Figure 1 and Figure 2. Thus, Lipid A was chosen as the lipid phase to prepare BCR-loaded lipid nanoparticles. Compritol 888 ATO is a glycerol ester of behenic acid-based solid lipid (C22) and has an amphiphilic property because of partial acylglycerols [24].

Different surfactants were screened for nanoparticle PS and PDI, such as Tween 20, Tween 80, Cremophore EL, and Pluronic F68 (a description of the effect of different types of surfactants is provided in the Supplementary Materials). Among other surfactants, the smallest PS (274.8 ± 4.5) was obtained with Tween 80 with low PDI (0.288 ± 0.0025). Thus, Tween 80 was chosen for the preparation of lipid nanoparticles. The PS and PDI decreased with Tween 80 compared to other surfactants used for screening. Tween 80 is a non-ionic surfactant that contains both hydrophobic (fatty acid chains) and hydrophilic units (ethylene oxide units) that offer amphiphilic properties [25]. Various studies show that nanoparticles with Tween 80 improve brain uptake of the coated molecule via receptor-mediated endocytosis, limiting drug delivery effectiveness to target sites and resulting in brain-targeted delivery, enhanced bioavailability, and improved therapeutic effect [26]. In addition, Tween 80 can inhibit P-glycoprotein, which causes drug efflux in the CNS [27]. It has also been reported that their coating avoids reticuloendothelial system (RES) uptake and prolongs the circulation time. Furthermore, Polysorbate 80 has hydrophilicity, nonionicity, biodegradability, and nontoxicity to cells at low concentrations and is easy to obtain [28].

### 2.2. Optimization of BCR-SLN and BCR-NLC

The optimization of BCR-SLN and BCR-NLC was carried out by applying the Box–Behnken Design (BBD). The variables A, B, and C had a significant effect on the U1, U2, and U3 of SLNs and V1, V2, and V3 of NLCs, as shown in Table 1, Table 2, Table 3 and Table 4.

Analysis of variance (ANOVA) was employed to test if the response at any level of the tested factor differs significantly from the other levels. If the Prob > F value is minimal (less than 0.05 is significant), it implies that the model terms influence the response. Furthermore, an ‘Adeq Precision’ ratio of 73.01, 27.87, and 30.63 for the responses U1, U2, and U3 revealed an adequate signal, indicating that this model may be utilized to navigate the design space since a ratio larger than 4 is acceptable. All batches of SLN showed particle sizes in the range from 224.16 nm to 314.91 nm, PDI 0.21 to 0.514, and EE 64.01 to 72.01%. SLN showed these values where, for the NLC, the particle sizes range from 196.5 nm to 272.5 nm, PDI 0.201 to 0.507, and EE 72.16 to 84.02.

The final quadratic model generated for BCR-SLN by the software is shown below in Equations (1)–(3).

U1 = + 257.22 + 24.99 A − 17.61 B − 10.92 C + 3.57 AB + 9.03 AC + 8.73 BC + 9.30 A^2^ + 8.78 B^2^ + 0.7185 C^2^ (1)

U2 = + 0.3002 + 0.0710 A − 0.0594 B − 0.0384 C + 0.0062 AB + 0.0288 AC + 0.0445 BC + 0.0429 A^2^ + 0.0431 B^2^ + 0.0162 C^2^ (2)

U3 = + 68.04 + 1.92 A + 0.1762 B + 1.15 C − 1.13 AB − 1.31 AC + 0.9000 BC + 1.32 A^2^ − 2.15 B^2^ + 0.8865 C^2^ (3)

The final quadratic model equations generated for BCR-NLC by the software are shown below in Equations (4)–(6).

V1 = + 228.28 + 22.93 A − 9.46 B − 9.01 C + 4.48 AB + 7.86 AC − 8.53 BC + 7.41 A^2^ + 7.64 B^2^ + 0.4702 C^2^ (4)

V2 = + 0.3614 + 0.0956 A − 0.0464 B − 0.0348 C − 0.0147 AB + 0.010 AC − 0.0155 BC − 0.0046 A^2^ − 0.0041 B^2^ − 0.0143 C^2^ (5)

V3 = + 77.60 − 3.91 A + 1.54 B + 0.4500 C + 0.3775 AB − 0.9700 AC − 0.4200 BC + 0.7252 A^2^ − 0.4648 B^2^ + 0.2178 C^2^ (6)

Equation (1) reveals that factors A have a positive impact on the PS, whereas B and C have a negative influence on the PS. As a result, increasing the solid lipid concentration increases the PS, whereas increasing the concentration of surfactant and homogenization speed lowers the PS. Equation (2) revealed that factor A has a positive impact on the PDI, whereas B and C have a negative impact on the PDI. As a result, increasing the amount of lipids would increase the PDI, whereas increasing the surfactant concentration and homogenization speed would lower the PDI. Finally, Equation (3) revealed that factors A, B, and C positively impact the EE. As a result, increasing the lipid concentration, surfactant concentration, and speed would result in a considerable increase in EE.

Equation (4) revealed that factor A has a positive impact on the PS, whereas B and C have a negative impact on the PS of NLC. As a result, increasing the solid lipid concentration increases the PS, whereas increasing the concentration of surfactant and homogenization speed lowers the PS. Equation (5) revealed that factor A has a positive impact on the PDI, whereas B and C have a negative impact on the PDI. As a result, decreasing the liquid lipid concentration would increase the PDI, whereas high surfactant concentration and homogenization speed would lower the PDI. Equation (6) reveals that factors B and C positively impact the EE, whereas factor A has a negative impact on the EE. As a result, increasing the surfactant concentration and speed would result in a considerable increase in EE, whereas increasing the concentration of solid lipids lowers the EE.

### 2.3. Influence of Independent Variables on Responses of BCR-SLN and BCR-NLC

#### 2.3.1. Perturbation Graphs for Optimization of BCR-SLN and BCR-NLC

**Figure 3:**
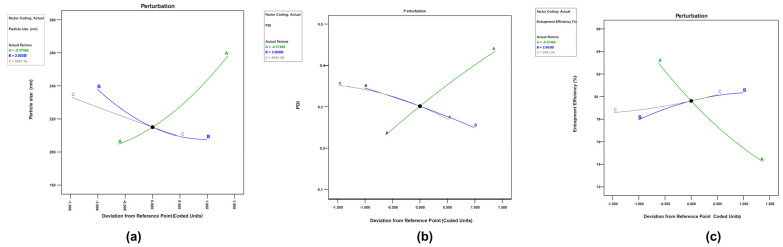
Perturbation graph for (**a**) U1 (PS), (**b**) U2 (PDI), and (**c**) U3 (EE).

**Figure 4:**
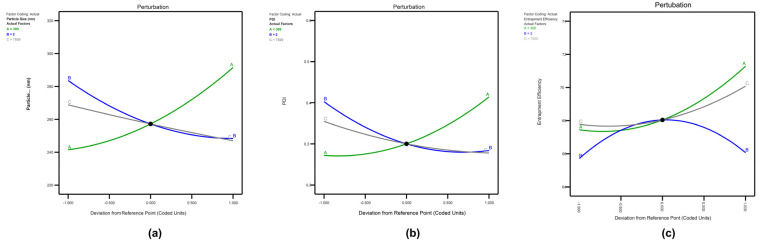
Perturbation graph for (**a**) V1 (PS), (**b**) V2 (PDI), and (**c**) V3 (EE).

The perturbation graph generated from the software was used to study the effect of factors on the response. Factors A and B display a steep curve for response U1 (Figure 3a), whereas factor C exhibits a modest slope. This implies that factor A, i.e., total lipid concentration, is the most critical factor determining the response’s result. An increased lipid content raises viscosity and surface tension, resulting in a higher PS. Factor A exhibits a high curvature, factor C, i.e., homogenization speed, has a slight but notable curvature, and factor B, i.e., surfactant concentration, exhibits a somewhat steep slope. As a result, the PS response was highly responsive to fluctuations in variable A. For outcome U2, factor A has a high slope (Figure 3b), factor B has a significant slope, and factor C has a slight bend relative to factor B. This suggests that the concentration of lipids and surfactants had the most significant influence on PDI. For the response U3 (Figure 3c), factor A showed a steep curvature compared to factors B and C, showing that increasing the lipid concentration enhanced the drug entrapment into the lipid matrix. In the case of NLC, factor A has a steeper curve than factors B and C for the response V1 (Figure 4a). This shows that factor A, i.e., total lipid concentration, is the most important independent variable determining the outcome of PS. Similarly, factor A had a sharp curve for the response V2 (Figure 4b), but factors B, i.e., surfactant concentration, and C, i.e., stirring speed, demonstrated a modest slope. This implies that when the lipid content increased, the PDI also increased. Factor A revealed a sharp curve for V3 (Figure 4c), indicating that an increase in solid lipid concentration reduces the EE.

#### 2.3.2. Response Surface Plots for Optimization of BCR-SLN

##### Effects of Variables on Responses

**Figure 5:**
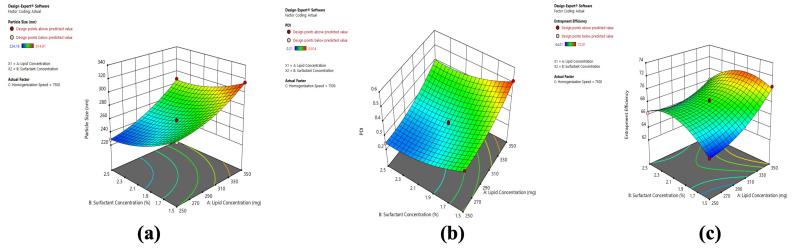
3D surface plots depicting the influence variables on (**a**) PS, (**b**) PDI, and (**c**) EE of BCR-SLN.

The color red or green (darker) indicates the highest response in the surface graphs, while blue is the lowest response. A decrease in PS with increasing C and B and higher PS with increasing A (Figure 5) can be observed. The image shows that higher PDI at the low level of B and C and lower at high B and C indicate PDI is decreasing with an increase in speed and surfactant concentration. Increased PDI was observed with increasing A. In the case of EE, an increased EE was found with increasing C, B, and A, from low to high levels.

#### 2.3.3. Response Surface Plots for Optimization of BCR-NLC

##### Effects of Variables on Responses

**Figure 6:**
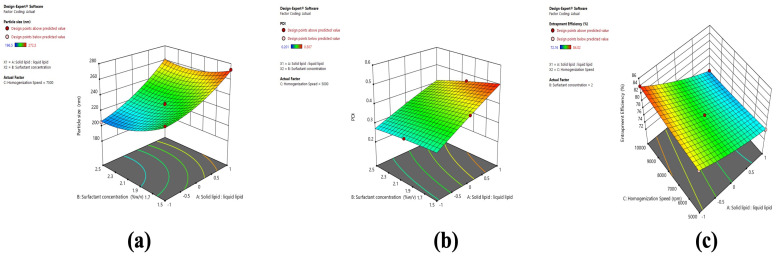
3D surface plots depicting the influence variables on (**a**) PS, (**b**) PDI, and (**c**) EE OF BCR-SLN.

It can be observed that PS decreases with increasing C and B, and PS increases with increasing A (Figure 6). The figure shows that higher PDI at a low level of B and C and lower PDI at high B and C indicates PDI is decreasing with an increase in speed and surfactant concentration (Figure 6). Increased PDI was observed with increasing A (Figure 6). In the case of EE (Figure 6), an increased EE was found with increasing C and B from low to high levels, whereas a decrease in EE was observed at a high level of A.

The response surface graph generated from the software was used to investigate the influence of variables on the responses U1, U2, U3, V1, V2, and V3. A significant decrease in PS was observed with increasing homogenization speed. Generated shear forces increase as a result of increased homogenization speed, leading to a decrease in emulsion droplet size and, as a result, a reduction in PS. It was observed that increasing B had an influence on the PS response. At the low-level prevalence, the green region was high, and at a higher level, the blue region was increasing, which indicates a decrease in PS with increasing B. Maryam et al. reported that a decrease in PS at higher surfactant concentrations reduces surface tension and stabilizes newly developed surfaces during homogenization [29]. The gradient inclination of solid lipid levels significantly increased the size. With increasing solid lipid levels, PS increases owing to vesicle accumulation, which causes aggregation and, eventually, size increases. The response surface graph for the effect of variables on the PDI of SLN and NLC shows that the increase in homogenization speed and surfactant concentration decreases the PDI, whereas increased liquid lipid concentration decreases the PDI. The response graph generated for the effect of the independent variables on EE of SLN shows increased homogenization speed, and surfactant concentration increases the EE. In contrast, increased liquid lipid concentration increases the EE. In the case of NLC, the graph shows that increased homogenization speed and surfactant concentration increased the EE, and increased solid lipid concentration decreased the EE. This could be attributed to the incorporation of oleic acid into Compritol; as the solid lipid: liquid lipid ratio increases from 70:30 to 90:10, the amount of oleic acid decreases. This causes a decrease in the EE of the NLC at a higher lipid ratio.

### 2.4. Optimization of Formulation

**Figure 7:**
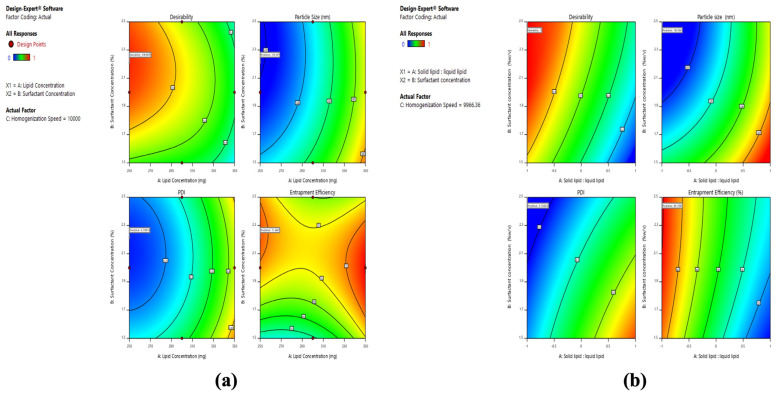
Desirability plots for optimization of (**a**) BCR-SLN and (**b**) BCR-NLC.

The BCR-SLN and BCR-NLC formulation, fulfilling the maximum requirement of response variables, was selected as an optimized formula based on the desirability value. The desirability values for BCR-SLN and BCR-NLC were found to be 0.9 and 1.0, respectively (Figure 7). The primary criteria for obtaining an optimal formulation of BCR-SLN and BCR-NLC were focused on achieving the smallest particle size, PDI, and high entrapment efficiency using the Design-Expert software’s (Version 11.1.2.0.) point prediction method. The run with the smallest size, PDI, and maximum EE was validated.

### 2.5. Evaluation of Optimized BCR-SLN and BCR-NLC Formulation

#### 2.5.1. Particle Size, PDI, and Entrapment Efficiency

**Figure 8:**
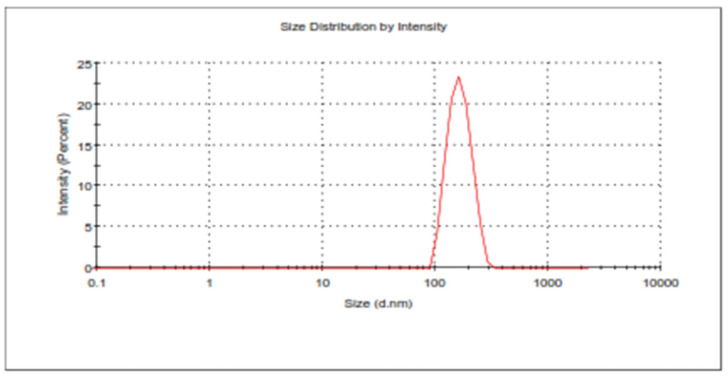
PS of optimized formulation BCR-SLN.

**Figure 9:**
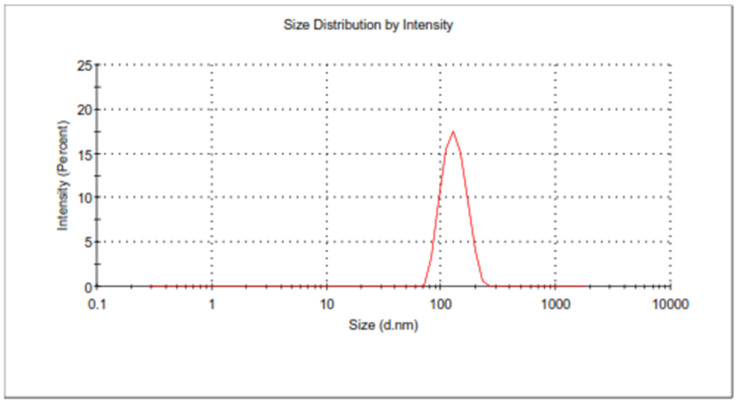
Particle size of optimized BCR-NLC.

The software predicted PS, PDI, and EE of 218.597 ± 1.64, 0.21 ± 0.013, and 71.34 ± 0.35, respectively, for BCR-SLN formulation and 182.006 ± 1.79, 0.153 ± 0.006, and 84.11 ± 0.14, respectively, for BCR-NLC. The obtained values for the BCR-SLN formulation showed a PS of 219.8 ± 1.3 nm, PDI of 0.22 ± 0.02 (Figure 8), and EE of 72.2 ± 0.5, and for BCR-NLC, the formulation showed a PS of 182.25 ± 2.2 nm, PDI of 0.16 ± 0.004 (Figure 9), and EE of 83.57 ± 1.8. They indicated that the observed values were in good agreement with the predicted values. Therefore, the solution with the smallest size, PDI, and maximum EE was chosen for further characterization based on the point prediction method.

#### 2.5.2. Microscopic Analysis by HR-TEM

**Figure 10:**
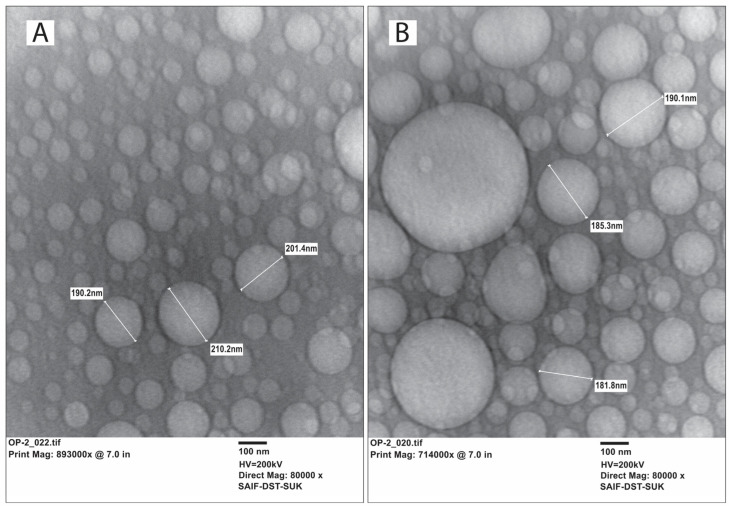
HR-transmission electron microscopy images of (**A**) BCR-SLN and (**B**) BCR-NLC.

The optimized SLN and NLC encapsulating BCR were subjected to morphological examination using transmission electron microscopy (TEM), as depicted in Figure 10A,B, respectively. The micrographs revealed predominantly round or oval shapes with a narrow size distribution, and the lipid formulations displayed a non-aggregated state. The particle sizes observed in the TEM images were consistent with the measurements obtained through dynamic light scattering (DLS) using a zeta-sizer. These observations confirm the successful development of a spherical nanosystem characterized by a size distribution in the nanosize range (<200 nm).

#### 2.5.3. DSC of Optimized Formulations

**Figure 11:**
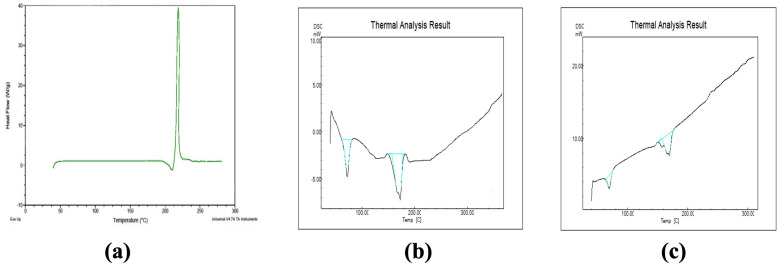
DSC of (**a**) BCR, (**b**) BCR-SLN, and (**c**) BCR-NLC.

DSC analysis is useful in assessing drug–lipid interactions and mixture behaviors. DSC plots for BCR-SLN and BCR-NLC are presented in Figure 11, showing a sharp exothermic peak at 214 °C (Figure 11a), which corresponds to its m.p. in the crystalline form. No peak of BCR was observed in the plot of the BCR-SLN (Figure 11b) and BCR-NLC (Figure 11c), which indicates the complete dissolution of BCR in the lipid matrix. A decrease in m.p. of the lipid nanoparticle was observed, which could be due to the drug incorporation in the lipid matrix. This results in an increase in the number of defects in the lipid crystal lattice [30], leading to the reduced m.p.

#### 2.5.4. In Vitro Release Studies

**Figure 12:**
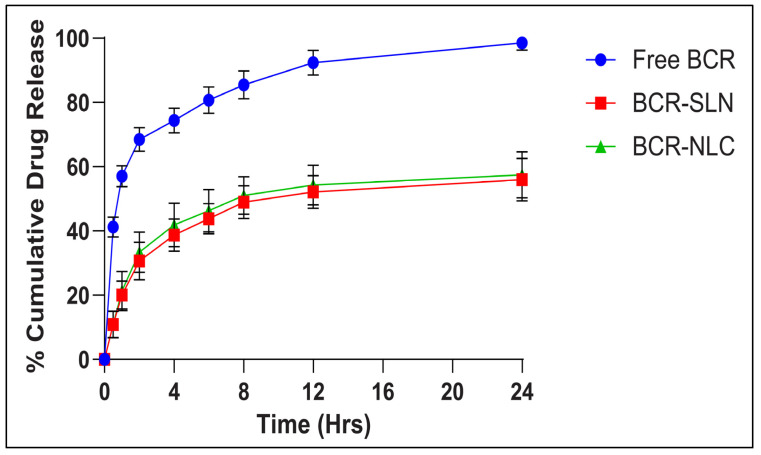
In vitro drug release study. Each value represents mean ± SD, and the experiment was performed in a replicate of three (*n* = 3).

The release profiles indicate that optimized BCR-SLN and BCR-NLC exhibited delayed drug release from the lipid matrix compared to free BCR (Figure 12). Degradation, erosion, and diffusion are the mechanisms of drug release from the SLN. The drug in the SLN is either incorporated in the matrix or present on the surface, and such a system can exhibit a biphasic drug release pattern (i.e., initial burst release of active moiety followed by the sustained drug release). Drugs bound to the surface of the SLN/NLC will dissociate from the nanoparticle and release immediately. Following that, the matrix can erode or degrade depending on the lipid nature, releasing the active moiety in a controlled manner. These results were in good agreement with the previously reported studies [31,32,33,34,35,36]. The initial burst release followed by sustained drug release can be attributed to the crystallization of Compritol, which initiates the formation of an inner lipid core. As solidification progresses, a solid drug solution may form around this lipid core, leading to a concentration of the drug on the outer surface. These findings align with those reported by Luan J et al., who also observed an initial burst release attributed to the dissolution, and the diffusion of the active compound adhered to the nanoparticle surface. Furthermore, they noted that the sustained release was a result of the degradation and erosion of the lipid matrix, with the drug eventually incorporated into the lipid core [36].

An increase in the amount of drug released from BCR-NLC compared to BCR-SLN, attributed to the incorporation of liquid lipid as described by Abousamra et al., increased drug release due to adherence of liquid lipid to the lipid matrix and due to the decrease in diffusion path length of the lipid matrix. This may be due to an increase in the amount of BCR in the outer shell of the nanoparticles due to the solubility of BCR in oleic acid, which increases the drug release rate [37]. The results were consistent with previous studies in which the incorporation of liquid lipid into a solid lipid creates structural defects in the solid lipid, resulting in a less ordered crystalline structure, hence enabling loaded drugs to release more easily, and also due to the decrease in PS, the increased surface area increases the release rate [38,39]. The obtained release data were fitted into various kinetic models like zero-order kinetics, first-order kinetics, the Higuchi model, and the Korsmeyer–Peppas model (Table 5). The release profile of both the SLN and the NLC were best fitted with the Korsmeyer–Peppas model based on the regression coefficient value. The n value > 0.5 indicates that the release from the SLN and the NLC is anomalous, i.e., the combination of erosion and diffusion [40,41].

The results obtained by applying various kinetic models suggested that the Korsmeyer–Peppas was the best-fit model. It implies that the release of the active moiety is caused by anomalous transport (n value between 0.5 and 1.0), suggesting that the release mechanism is unknown, or that more than one type of release phenomenon may be involved.

### 2.6. Storage Stability Studies

The stability study result indicated a slight increase in PS of SLN and NLCs after six months of storage (Table 6). Although the PS of both the BCR-SLN and BCR-NLC had a certain degree of growth, the lowest increase was observed at 4 °C rather than at 25 °C, and the change in PS was least with BCR-NLC compared to BCR-SLN. The results show that as the duration of storage increased, NLCs could be more stable than SLN. The smallest PS with NLC could be attributed to the incorporation of liquid lipid to solid lipid, causing crystal order disturbance to supply enough space to accommodate drug molecules. The PDI results during the storage showed a smaller size distribution of BCR NLC than BCR SLN. The results of EE of SLN were higher than NLC. There was a significant decrease in the EE of BCR-NLC compared to BCR-SLN. A reduction in EE of SLN might be due to drug expulsion during storage. This could be attributed to increased drug loading capacity in NLC due to imperfect crystalline structure, and it also has the potential to prevent drug expulsion by preventing lipid crystallization during manufacture and storage, which are in good agreement with the literature [42]. The blend of liquid lipid and solid lipid increases the solubility of the drug, and imperfections in the crystalline structure result in more space for the drug in the lipid matrix, which improves the EE of the drug in the NLC.

### 2.7. In Vitro Cytotoxicity Studies by MTT Assay

**Figure 13:**
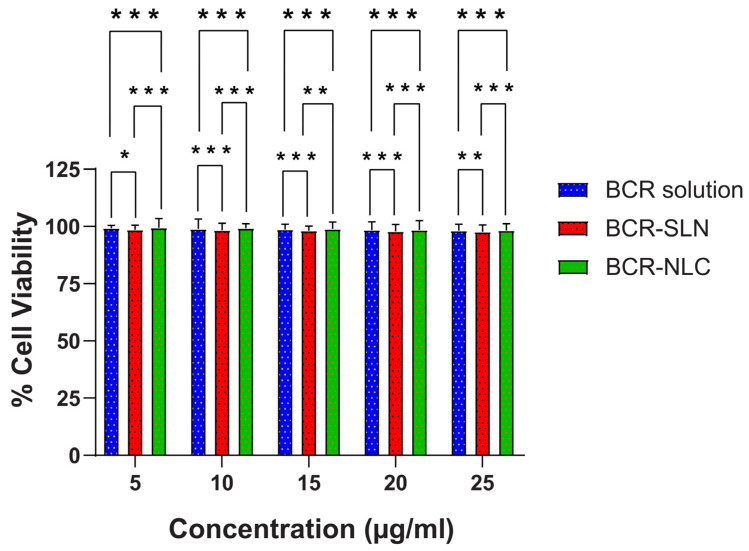
% cell viability of BCR, BCR-SLN, and BCR-NLC. (Data expressed as mean ± SD, *n* = 3; * *p* < 0.05, ** *p* < 0.01, *** *p* < 0.001).

Different formulation factors, such as surface charge and surfactant concentration, might cause neurotoxicity. Hence, cytotoxicity investigations of the nanoformulations are strongly recommended. The MTT test, which provides information regarding cell metabolism activities, is widely employed. Cellular metabolic activity reduction has been utilized as an indication of damaged cells. In this work, all formulations were tested for 24 h in a concentration range of 5 µM to 25 µM. Figure 13 depicts the % cell viability data. The results show nearly a hundred percent cell viability of SLN, NLC, and the pure drug, indicating that the nanoformulations are non-toxic to the neuronal cells and, hence, are safe for brain delivery.

### 2.8. Pharmacokinetic and Brain Distribution Kinetic Studies

**Figure 14:**
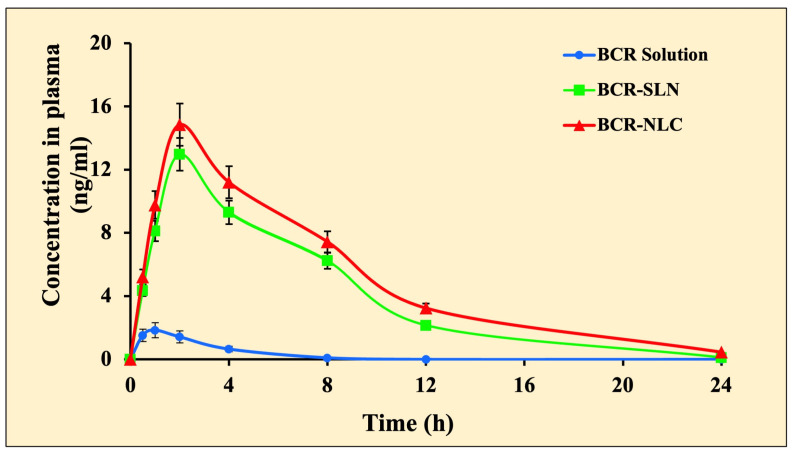
Concentration versus time profile of plasma BCR-solution, BCR-SLN, and BCR-NLC. Each value represents mean ± SD, and the experiment was performed in a replicate of three (*n* = 3).

**Figure 15:**
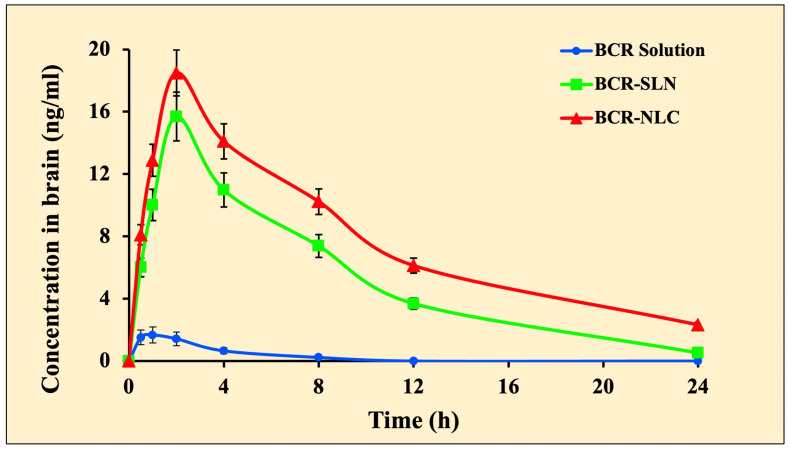
Concentration versus time profile of brain BCR-solution, BCR-SLN, and BCR-NLC. Each value represents mean ± SD, and the experiment was performed in a replicate of three (*n* = 3).

In the drug solution, the area under the curve (AUC_0-t_) in plasma was less than in the other groups (Table 7, Figure 14 and Figure 15). Compared to the BCR solution, BRC-SLNs and BRC-NLCs demonstrated a significant increase in plasma and brain concentration of BCR. In vivo findings showed that BCR-NLCs increased the absorption of plasma BCR and brain BCR by more than 1.9 times compared to BCR-SLNs. During plasma pharmacokinetic and brain distribution kinetic studies, the lipid nanoparticles showed a detectable amount of the BCR in the plasma, whereas in the case of a drug solution, the level was not detectable at 8 h. Solution-treated animal brains did not show any drug distribution in the brain at 8 h. On the other hand, lipid nanoparticles showed a detectable amount of the drug in the brain.

Various pharmacokinetic parameters such as peak plasma concentration (Cmax), time to peak plasma concentration (Tmax), area under the curve (AUC total), and biological half-life (t1/2) of BCR following oral administration of the BCR solution, optimized BCR-SLN, and BCR-NLC formulation were calculated and compared. The study indicated that both the BCR-SLN and BCR-NLC formulations improve the drug’s oral bioavailability compared to the BCR Solution. The peak plasma concentration value was higher for the BCR-SLN and BCR-NLC formulations than for the BCR solution following the oral administration. An increase in the time it takes to reach the maximum plasma concentration for SLN and NLC indicates a slower absorption of lipid nanoparticles than the plain drug. The results indicated that lipid-based delivery systems might bypass the hepatic first-pass route. The AUC expresses that the amount of drug that comes into systemic circulation after administration was considerably higher for the BCR-SLN and BCR-NLC formulations than the BCR solution form. An increase in half-life suggested the prolonged release of the drug. The significantly slower elimination rate of the BCR-SLN and BCR-NLC formulations than the drug solution could explain the considerably longer retention of the drug. It is hypothesized that the enhanced bioavailability could be attributed to the slow elimination rate, increased residence time, smaller PS, the solubility of the drug in the lipid, and the protection of the drug from chemical and enzymatic degradation [43] could be due to the slower degradation rate of long-chain fatty acids in the lipid matrix that results in slow release from the lipid matrix [44]. Lipid nanoparticles have been shown to improve BCR bioavailability via lymphatic absorption, which has numerous advantages over portal blood absorption, including reduced hepatic first-pass metabolism and, hence, increased bioavailability. The lipids used in the lipid-based nanoparticles have the same structure as fat-rich foods. This could cause bile secretion, and since the interaction between the drug and bile salt leads to the formation of mixed micelles, the NLCs enter the lymphatic vessels and avoid the first-pass effect [45]. In addition, enhanced bioavailability is probably due to the prolonged drug release through avoiding the first-pass effect [46]. Furthermore, an increase in bioavailability of NLC than SLN might be related to its PS. The delayed Tmax can be due to the encapsulation of the drug in the lipid matrix, which results in the slow and sustained release from the lipid matrix.

In the case of brain distribution studies Cmax, Tmax, AUC total, and t1/2 of BCR following oral administration of the drug solution, optimized BCR-SLN, and BCR-NLC formulation were calculated and compared. The study indicated that the BCR-SLN and BCR-NLC formulations improve the drug’s oral bioavailability compared to the BCR solution. The peak plasma concentration value was higher for the BCR-SLN and BCR-NLC formulations than for the BCR solution following oral administration. An increase in the time it takes to reach the maximum concentration for SLN and NLC suggests the sustained release of the drug. The considerably slower elimination rate from the BCR-SLN and BCR-NLC formulations compared to the drug solution could explain the significantly prolonged drug retention. The AUC of the BCR-SLN and BCR-NLC formulations was significantly higher than the BCR solution form, suggesting a longer duration of action. Solution-treated animal brains did not show any drug distribution in the brain at 8 h. On the other hand, lipid nanoparticles showed a detectable amount of the drug in the brain. The solubilization of endothelial cell membrane lipids and membrane fluidization due to Tween 80’s surfactant effects, endocytosis of intact lipid nanoparticles from the blood–brain barrier, and longer duration of nanoparticles remaining in contact with BBB cells due to enhanced blood bioavailability and increased plasma concentration could be the possible mechanism for higher peak concentration and improved bioavailability in the brain. Tween 80 improves the absorption of SLN molecules from the BBB endothelium’s LDL receptors. Tween 80 is used in SLN formulations to facilitate the drug’s passage across LDL receptors and apolipoprotein E to the brain [47]. Lipid nanoparticles’ small PS, high surface area, and lipophilic nature enhance contact time with the BBB and promote their transit into the brain, resulting in a drug concentration gradient [48]. Polysorbate-80 has been demonstrated to increase the amount of apolipoprotein E (apoE) adsorbed on nanoparticle surfaces, and these apoE-enriched nanoparticles are more likely to take advantage of LDL receptor-mediated endocytosis in brain endothelial cells [8].

## 3. Materials and Methods

### 3.1. Materials

Bromocriptine was purchased from Clearsynth Labs Ltd., Mumbai, India. Compritol ATO 888 was provided by Gattefosse Mumbai, India as a gift sample. Tween 80 and Oleic acid were obtained from Loba Chemie, Mumbai, India. The dialysis membrane was supplied from HiMedia Labs, Mumbai, India.

### 3.2. Screening of Solid Lipid and Liquid Lipid

#### 3.2.1. Solubility Study

One of the key parameters affecting drug-loading capacity in lipids is the drug’s lipid solubility. Various lipids were evaluated for their ability to dissolve BCR. Briefly, 5 mg of BCR was taken in wide-mouth screw-capped bottles. Separately, the solid lipids were melted by heating to a temperature above their melting point. Further, the lipid was gradually added in portions to the bottle containing BCR with continuous stirring. The quantity of molten lipids required to form a clear solution was determined. The study’s endpoint was the formation of a clear solution, which indicates the drug’s saturation solubility in the lipid. The lipid was chosen based on the maximum solubility of the drug in the lipid [49,50].

#### 3.2.2. Partitioning Behaviour of Bromocriptine

Five milligrams of BCR was dispersed into a blend of melted lipids and hot distilled water. The mixture was shaken for half an hour, cooled, and then centrifuged. The aqueous phase was filtered using a membrane filter and analyzed by the HPLC method (Shimadzu LC 2010A HT, Kyoto, Japan) to determine the drug concentration in the aqueous phase to study its partitioning behaviour with different lipids [51,52,53].

#### 3.2.3. Screening of Liquid Lipid

In a preliminary lipid screening study, Compritol was chosen as the lipid for formulating Solid Lipid Nanoparticles (SLN). For the formulation of Nanostructured Lipid Carriers (NLC), a selection process was undertaken to identify suitable liquid lipids. This involved assessing the solubility of the drug in different liquid lipids, namely Labrasol, Lauroglycol FCC, Captex 300, coconut oil, oleic acid, and sunflower oil. Each liquid lipid was evaluated to determine its efficacy in solubilizing the drug, thus aiding in the selection of the most appropriate candidate for the NLC formulation.

#### 3.2.4. Effect of Solid Lipid: Liquid Lipid Ratio

Various ratios, ranging from 90:10 to 70:30, were utilized in the formulation of NLC. During this process, the concentration of the surfactant was fine-tuned to optimize factors such as entrapment efficiency, PDI, and miscibility. It is important to note that parameters such as lipid concentration and homogenization speed remained consistent throughout this optimization phase. By adjusting the surfactant concentration within these ratios, we aimed to achieve the ideal balance of properties necessary for effective drug delivery within the NLC system.

### 3.3. Preparation of BCR-SLNs and NLCs

BCR-SLNs were prepared using the High-Pressure Homogenization method followed by ultra-sonication. Briefly, BCR was weighted accurately and incorporated into Compritol 888 ATO previously melted at 80 °C (mixture of solid and liquid lipid in case of NLC). Tween 80 was dispersed in distilled water to prepare an aqueous surfactant solution and boiled to near 80 °C. The hot aqueous Tween 80 solutions were added into the hot lipid phase under continuous stirring (REMI 1-ML Electromagnetic stirrer, REMI Instruments, Mumbai, India), and homogenization (PT1600E, Kinematica Polytron, Lucerne, Switzerland) was carried out. The obtained *o*/*w* nanoemulsion was left to cool to room temperature to solidify and produce lipid nanoparticles [54,55,56]. Then the method developed and optimized in our laboratory was used for lyophilization (Alpha1-2 LD Plus, Martin Christ, Osterode am Harz, Germany) using 5% *w*/*v* of mannitol as a cryoprotectant. The freeze-dried sample was stored for future studies [57].

### 3.4. Optimization of SLNs and NLCs by Box Behnken Design

A scientific and systemic experimental design approach was employed to investigate the impact of independent variables on dependent variables. A 17-run experiment with five centre points of Box–Behnken design utilizing the Design Expert^®^ software (Version 11.1.2.0.) was employed for the optimization of BCR-SLN and BCR-NLC. The effect of the independent variables on the dependent responses was optimized using a three-factor, three-level BBD. The selected variables were examined at three levels: low (–1), medium (0), and high (+1), as shown in Table 8 and Table 9. These variables are lipid concentration (A), surfactant concentration (B), and homogenization speed (C). Various batches were produced, and the data were entered into the design expert software. The key factor in selecting the optimized formula was the least PS, PDI, and maximum EE.

### 3.5. Characterization of Prepared Nanoparticles

#### 3.5.1. Nanoparticle Size Analysis

The prepared lipid nanoparticles were evaluated for PS and PDI by dynamic light scattering (DLS), employing a Malvern instrument (DTS Ver.5.10, Malvern Instruments Ltd., Malvern, UK) at 25 ± 1 °C. The samples were mixed with milli-Q water before being analyzed and were measured three times [58,59].

#### 3.5.2. Entrapment Efficiency

EE was estimated by centrifugation of BCR-SLN and NLC at 15,000 rpm for 30 min. The supernatant was separated and suitably diluted to determine the amount of free BCR in the supernatant using an HPLC (Shimadzu LC 2010A HT, Kyoto, Japan), using the following equation [60].

(7) $$Entrapment\;efficiency\;\%=\left[\frac{\left(Total\;drug-drug\;in\;the\;supernatant\right)}{Total\;drug}\right]\times 100$$

#### 3.5.3. High-Resolution Transmission Electron Microscopy (HR-TEM) Analysis

HR-TEM (JEOL, JM 2100, Tokyo, Japan) was employed to investigate the surface morphology of SLN and NLC encapsulating BCR. A copper grid was used to hold a small quantity of the lipid formulation, which was then dried for approximately 3 min before being introduced into the microscope for imaging purposes.

#### 3.5.4. Crystallinity Studies Using DSC

The purity of the sample and compatibility between the drug and components of nanoparticle formulation were studied using DSC (DSC 60, Shimadzu, Kyoto, Japan). DSC thermograms for different samples were obtained at a heating rate of 10 °C in the range of 30–300 °C under the nitrogen purge of 50 mL/min. An empty pan was utilized as a reference, and the sample was taken in a standard aluminium sample pan under nitrogen purge and then analyzed [61].

#### 3.5.5. In Vitro Release Studies

Drug release studies were carried out in vitro using the dialysis bag technique. The dialysis membrane used had a pore size of 2.4 nm and a molecular weight cutoff of 12,000–14,000 nm. Prior to the experiment, the membranes were activated by soaking them in a buffer solution for approximately 24 h. The sink condition was maintained while experimenting. In a double-sided, opening glass tube, the BCR solution and the nanoparticle were taken separately and fastened at one end with an activated dialysis membrane that had been soaked in buffer solution for the entire night. This glass tube was maintained at 37 ± 5 °C with a magnetic stirrer running at 50 rpm, and it contained 200 mL of pH 7.4 phosphate buffer (release media). To maintain the conditions in a sinking state, 5 mL of an aliquot was taken out of the medium at 0.5, 1, 2, 4, 6, 8, 12 h, and 24 h. The aliquot was then replaced with the same volume of freshly prepared buffer. The samples were assayed by employing the HPLC method (Shimadzu LC 2010A HT, Kyoto, Japan), and the cumulative percentage release of drugs versus time profile was plotted. The experiments were performed in triplicate and the obtained results were fitted to zero-order kinetics, first-order kinetics, the Higuchi model, and the Korsmeyer–Peppas kinetic models [62,63,64].

### 3.6. In Vitro Cytotoxicity Study

The SH-SY5Y, a human neuroblastoma cell line was employed to perform cytotoxicity studies. The cell was maintained in DMEM low glucose supplemented with 15% (*v*/*v*) FBS and 100 µg/mL penicillin-streptomycin and DMEM at 37 °C and 5% CO_2_. Cells were subcultured frequently using trypsin, and the cells were suspended in a 10% growth medium. Cells were seeded in 96-well tissue culture plates and incubated. MTT (5 mg/mL) was prepared and filtered through a 0.22 µm filter to ensure sterility, and then different concentrations of test and standard were prepared in 5% DMEM and filtered through a 0.22 mm Millipore syringe. After 24 h, 30 µL of MTT solution was added to each well. The plates were rotated for 5 min to distribute evenly and incubated at 37 °C, 5% CO_2_ for 4 h. The formazan crystals were dissolved by adding 100 mL of DMSO and absorbance was measured at 540 nm to determine the % viability of the cells using the following formula [65].

(8) $$\%\;of\;viability=\left[\frac{Mean\;optical\;density\;of\;sample}{Mean\;optical\;density\;of\;control}\right]\times 100$$

### 3.7. Stability Study

The stability assessment of the optimized BCR-SLN and NLC formulations involved storing the samples in a stability chamber (GMP-SC400L, Kesar Control Systems, Ahmedabad, India) for a period of up to 6 months. These samples were kept at controlled storage temperatures of 4 ± 1 °C and 25 ± 1 °C throughout the study duration. At specific intervals, namely 0 days, 1 month, 3 months, and 6 months, the BCR-SLN and NLC formulations were evaluated for PS, PDI, and EE. This systematic approach allowed for a comprehensive examination of the formulations’ stability under varying storage conditions over an extended period.

### 3.8. In Vivo Studies

#### 3.8.1. Experimental Animals and In Vivo Pharmacokinetic and Brain Distribution Kinetic Study of BCR-SLN and BCR-NLC

The pharmacokinetics and brain distribution kinetic study were approved by the Institutional Animal Ethics Committee (IAEC), JSS College of Pharmacy, Ooty, India (Protocol approval No. JSSCP/OT/IAEC/04/2021-22). In vivo experiments were performed on healthy adult Wistar rats (200–250 g). The animals were kept in cages in a controlled environment at a temperature of 25 ± 1 °C and 45–55% relative humidity (Rh). The rats were divided into three groups of 3 in each. Rats were fed a standard laboratory diet and water ad libitum. Pure BCR solution, BCR-SLN, and BCR-NLC were administered to Groups I, II, and III, respectively, via 18 mm gauge oral gavage. In brief, around 0.5 mL of blood samples were collected via retro-orbital puncture at various time intervals (0.5, 1.0, 2.0, 4.0, and 8.0 h) into a centrifuge tube containing anticoagulant followed by centrifugation at 5000 rpm for 15 min, and separated plasma samples were stored at –20 °C ± 2 °C until further analysis. Three animals from each group were sacrificed immediately after blood collection at each time point (0.5, 1.0, 2.0, 4.0, and 8.0), and their brains were surgically excised and washed in PBS, pH 7.4, to remove the blood and connective tissue. The brain tissues were homogenized with 0.1 M phosphate buffer (pH 7.4) (1:1.5) at 5000 rpm for 15 min. The supernatant was then stored at −200 C until further analysis by the HPLC method to determine the quantity of BCR in the brain homogenate. Based on USFDA guidelines, the dosage was determined using an animal formula by multiplying the human dose (mg/kg) by the Km ratio, which was found to be 0.5 mg/kg.

#### 3.8.2. Analysis of BCR in Brain Samples

The concentration of BCR in the brain sample was measured with the RP-HPLC method (Shimadzu LC 2010A HT, Kyoto, Japan) using the modified procedure reported by Sita et al. [66]. The chromatographic separation of BCR and lurasidone (internal standard) was effectively achieved on Princeton^®^ C18 Column (250 × 4.6 mm, i.d. 5 µm) with a flow rate of 1 mL/min, injection volume of 20 μL, and the wavelength of estimation for BCR was fixed at 302 nm. The mobile phase used for the chromatographic separation was acetonitrile and ammonium acetate in the ratio of 60:40. Different concentrations of BCR solutions were prepared using the standard stock solution to prepare the calibration curve. The linearity range was from 2 to 10 ng/mL. Briefly, heparinized plasma samples (180 μL), brain homogenate spiked with BCR solution (10 μL of various concentrations), and a fixed concentration of the internal standard stock solution (10 μL) were analyzed using the HPLC. The calibration curves of BCR were constructed with brain samples by plotting concentrations against the respective peak area ratios of BCR and IS. Detailed data of the analytical method development and validation of the BCR by RP-HPLC method are provided in the Supplementary Materials. The homogenized brain samples were analyzed to determine the BCR concentration by RP-HPLC using the salt-assisted liquid–liquid extraction (SALLE method). Briefly, the internal standard stock solution (10 μL) was added to plasma and brain homogenates (190 μL) and vortexed. ACN was added under vortex and then NaCl was added to cause the precipitation of plasma components followed by centrifugation for 15 min at 5000 rpm. The supernatant was separated and analyzed with the HPLC system.

#### 3.8.3. Statistical Analysis

GraphPad Prism 8.0.2 software (GraphPad Software Inc., San Diego, CA, USA) was used to statistically analyze all the recorded research data. For independent experiments, the results are reported as the mean ± SD (*n* = 3 to 15). One-way ANOVA with post-hoc analysis implying Dunnett’s Multiple Comparisons Test was used to assess differences amongst the groups. *p* < 0.05 was considered to be a statistically significant difference among all the groups [ns—non-significant, * *p* < 0.05, ** *p* < 0.01, *** *p* < 0.001, and **** *p* < 0.0001 (in comparison to the control group)].

## 4. Conclusions and Future Directions

The utilization of nanoparticulate drug delivery systems represents a groundbreaking frontier in pharmacology, offering a highly promising avenue for delivering therapeutics with unprecedented precision and efficacy. This approach holds the potential to revolutionize medication delivery by enabling more targeted, selective, and potent treatment options while mitigating the adverse effects commonly associated with traditional drug administration. In light of these advancements, our research endeavors were aimed at designing and meticulously characterizing a nanoparticulate delivery system specifically tailored for BCR, an anti-Parkinson’s drug crucial for managing Parkinson’s disease (PD). The overarching goal was to engineer a delivery mechanism that not only prolongs the drug’s efficacy but also facilitates its enhanced permeation into the brain, where its therapeutic effects are most needed.

To achieve this, we employed a sophisticated methodology known as BBD to optimize two distinct formulations: BCR-loaded SLNs and BCR-loaded NLCs. By utilizing Design of Experiments (DOE), we significantly streamlined our experimental process, reducing costs and saving valuable time without compromising the quality of our results. Upon optimization, our formulations exhibited a distinct biphasic release pattern, indicative of their controlled and sustained drug release profiles. Importantly, extensive cytotoxicity studies conducted on SHSY5Y cell lines, as well as hemocompatibility assessments, underscored the safety and biocompatibility of the developed lipid nanoparticles, further validating their potential for clinical translation. Crucially, our in vivo experiments revealed a remarkable enhancement in both plasma and brain concentration profiles of BCR following the administration of the optimized BCR-SLNs and BCR-NLCs. Particularly noteworthy was the finding that BCR-NLCs outperformed BCR-SLNs, exhibiting a more than 1.9-fold increase in the area under the concentration–time curve (AUC) for both plasma and brain concentrations of BCR. This compelling evidence underscores the tremendous potential of our lipid nanoparticle-based approach in significantly augmenting the oral bioavailability and BBB penetration of BCR, thereby offering a promising avenue for the management of PD. These groundbreaking findings underscore the viability of lipid nanoparticles as an efficacious carrier system for enhancing the oral delivery of therapeutics targeting the CNS, particularly for drugs characterized by poor aqueous solubility. Moreover, our study underscores the transformative potential of optimizing NLCs as a versatile drug delivery platform, opening up exciting possibilities for the treatment of CNS disorders with poorly water-soluble drugs.

Moving forward, continued research and exploration in this realm hold immense promise in advancing the frontiers of pharmacotherapy, offering hope for the development of novel treatment modalities capable of addressing the unmet clinical needs of patients suffering from a myriad of CNS disorders. Firstly, fine-tuning formulations to optimize stability, drug loading, and release kinetics can enhance therapeutic outcomes. Targeted delivery strategies, such as ligand-mediated targeting or stimuli-responsive systems, offer the promise of even greater precision in drug delivery, minimizing off-target effects. Additionally, exploring combination therapies within the same nanoparticulate delivery system could provide synergistic benefits for treating PD and other CNS disorders.

## Figures and Tables

**Table 1:** Factor level and observed responses of BCR-SLN in BBD.

Run	A	B	C	PS (U1)	PDI (U2)	EE (U3)
	mg	%	RPM	nm		%
1	250	2	5000	262.32 ± 12.31	0.364 ± 0.025	65.87 ± 1.10
2	300	2.5	5000	251.78 ± 10.28	0.289 ± 0.022	65.24 ± 0.96
3	250	1.5	7500	270.1 ± 12.02	0.387 ± 0.039	64.01±1.45
4	300	2	7500	257.78 ± 11.89	0.302 ± 0.042	67.91 ± 2.22
5	300	1.5	10,000	264.21 ± 8.12	0.341 ± 0.031	66.52 ± 1.56
6	350	1.5	7500	314.91 ± 10.58	0.514 ± 0.035	70.42 ± 0.87
7	350	2.5	7500	287.64 ± 11.26	0.398 ± 0.041	68.17 ± 1.34
8	300	2	7500	258.54 ± 9.44	0.297 ± 0.024	67.74 ± 1.89
9	350	2	10,000	290.21 ± 8.06	0.412 ± 0.032	72.01 ± 2.31
10	250	2.5	7500	228.54 ± 7.25	0.246 ± 0.018	66.27 ± 1.89
11	300	2.5	10,000	245.65 ± 11.21	0.321 ± 0.042	69.02 ± 3.45
12	250	2	10,000	224.16 ± 8.51	0.21 ± 0.016	71.12 ± 2.19
13	350	2	5000	292.24 ± 10.88	0.451 ± 0.019	72.01 ± 1.49
14	300	2	7500	256.12 ± 9.11	0.314 ± 0.030	68.34 ± 2.37
15	300	2	7500	256.64 ± 12.25	0.291 ± 0.025	68.21 ± 1.12
16	300	1.5	5000	305.24 ± 13.48	0.487 ± 0.031	66.34 ± 1.87
17	350	2	7500	257.01 ± 11.87	0.297 ± 0.015	68.01 ± 2.18

The findings are expressed as mean ± SD; *n* = 3.

**Table 2:** Regression terms for responses of BCR-SLN.

	R^2^ Value	Predicted R^2^	Adjusted R^2^
U1	0.9981	0.9747	0.9956
U2	0.9885	0.8545	0.9737
U3	0.9896	0.8726	0.9763

**Table 3:** Factor level and observed responses of BCR-NLC in BBD.

Run	A	B	C	PS (V1)	PDI (V2)	EE (V3)
		(% *w*/*v*)	(rpm)	(nm)		(%)
1	90:10	2.5	7500	259.6 ± 9.51	0.391 ± 0.040	75.87 ± 4.4
2	80:20	2	7500	226.8 ± 8.50	0.364 ± 0.022	77.65 ± 2.9
3	80:20	2.5	10,000	210.5 ± 6.55	0.245 ± 0.021	78.91 ± 3.1
4	70:30	2	10,000	196.5 ± 8.32	0.201 ± 0.018	84.02 ± 2.1
5	70:30	2.5	7500	205.2 ± 7.61	0.228 ± 0.032	82.81 ± 5.2
6	80:20	2	7500	228.01 ± 8.54	0.349 ± 0.025	77.54 ± 2.5
7	90:10	2	10,000	258.5 ± 5.87	0.411 ± 0.031	74.13 ± 3.5
8	80:20	1.5	10,000	243.54 ± 7.77	0.375 ± 0.035	76.54 ± 1.8
9	70:30	1.5	7500	236 ± 9.01	0.285 ± 0.021	80.61 ± 2.2
10	90:10	1.5	7500	272.5 ± 8.53	0.507 ± 0.067	72.16 ± 2.8
11	90:10	2	5000	260.1 ± 9.14	0.464 ± 0.051	75.01 ± 1.9
12	80:20	2	7500	228.4 ± 7.53	0.368 ± 0.026	77.49 ± 2.3
13	80:20	2.5	5000	246.3 ± 8.22	0.342 ± 0.031	79.01 ± 2.4
14	70:30	2	5000	229.54 ± 10.31	0.294 ± 0.026	81.02 ± 2.0
15	80:20	2	7500	228.7 ± 7.84	0.365 ± 0.023	77.68 ± 2.5
16	80:20	1.5	5000	245.21 ± 12.31	0.41 ± 0.040	74.96 ± 1.8
17	80:20	2	7500	229.5 ± 8.10	0.361 ± 0.024	77.65 ± 2.4

The findings are expressed as mean ± SD; *n* = 3.

**Table 4:** Regression terms for responses of BCR-NLC.

	R^2^ Value	Predicted R^2^	Adjusted R^2^
V1	0.9966	0.9548	0.9923
V2	0.9969	0.9804	0.9929
V3	0.9991	0.9874	0.9978

**Table 5:** Dissolution model kinetics by fitting dissolution data of BCR-SLN and BCR-NLC.

	Zero-Order	First Order	Higuchi	Korsmeyer-Peppas	
	R^2^	R^2^	R^2^	R^2^	n
SLN	0.7572	0.8322	0.9449	0.9516	0.54
NLC	0.6738	0.7792	0.9037	0.9238	0.55

**Table 6:** Storage stability studies of optimized BCR-SLN and BCR-NLC formulations.

Formulation	Temp.	Parameters	Sample Interval (Months)			
			0	1	3	6
BCR SLN	4 °C	PS (nm)	219.21 ± 1.3	226.2 ± 7.6	232 ± 5.6	241.4 ± 6.1
		PDI	0.22 ± 0.02	0.25 ± 0.042	0.31 ± 0.038	0.42 ± 0.062
		EE (%)	72.2 ± 1.20	67.78 ± 0.74	61.24 ± 0.48	56.87 ± 0.75
	25 °C	PS (nm)	219.21 ± 1.3	229.8 ± 2.9	236 ± 6.6	249.9 ± 6.4
		PDI	0.22 ± 0.02	0.29 ± 0.043	0.31 ± 0.023	0.45 ± 0.087
		EE (%)	71.12 ± 1.20	68.01 ± 0.81	65.21 ± 0.91	52.24 ± 1.68
BCR NLC	4 °C	PS (nm)	182.87 ± 2.2	184.54 ± 3.3	186 ± 3.6	189.79 ± 4.9
		PDI	0.16 ± 0.004	0.16 ± 0.43	0.18 ± 0.23	0.19 ± 0.87
		EE (%)	83.57 ± 1.2	81.96 ± 0.89	80.58 ± 0.67	79.02 ± 0.47
	25 °C	PS (nm)	182.87 ± 2.2	184.7 ± 2.8	188.8 ± 4.8	191.2 ± 4.6
		PDI	0.16 ± 0.004	0.17 ± 0.15	0.18 ± 0.46	0.20 ± 0.44
		EE (%)	83.57 ± 1.2	81.84 ± 0.87	79.04 ± 0.69	77.88 ± 0.24

The findings are expressed as mean ± SD; *n* = 3.

**Table 7:** Plasma pharmacokinetics and brain distribution kinetic studies data.

	BCR Solution		BCR-SLN		BCR-NLC	
	BRAIN	PLASMA	BRAIN	PLASMA	BRAIN	PLASMA
Cmax (ng/mL)	1.66 ± 0.008	1.84 ± 0.01	15.69 ± 1.16	12.96 ± 0.29	18.49 ± 0.953	14.84 ± 0.29
T_max_ (h)	1.0	1.0	2.00	2.00	2.00	2.00
T_1/2_	1.60 ± 0.16	2.17 ± 0.02	6.11 ± 0.49	5.79 ± 0.31	7.12 ± 0.16	6.87 ± 0.63
K_e_ (h^−1^)	0.44 ± 0.04	0.32 ± 0.004	0.11 ± 0.009	0.12 ± 0.006	0.09 ± 0.002	0.10 ± 0.009
AUC (ng h/mL)	5.16 ± 0.28	6.50 ± 0.09	84.13 ± 5.98	69.83 ± 3.06	103.37 ± 3.57	80.28 ± 2.57
AUC	5.37 ± 0.35	7.14 ± 0.12	150.96 ± 11.3	121.67 ± 8.09	205.98 ± 5.41	156.59 ± 10.6

The findings are expressed as mean ± SD; *n* = 3.

**Table 8:** Coded independent variables for optimization of BCR-SLN in BBD.

Independent Variables	Levels Used		
	−1	0	1
Solid lipid concentration (mg)	250	300	350
Surfactant concentration (% *w*/*v*)	1.5	2.0	2.5
Homogenization Speed (rpm)	5000	7500	10,000
**Dependent variables**			
PS (nm) (U1)	Minimum		
PDI (U2)	Minimum		
EE (%) (U3)	Maximum		

**Table 9:** Coded independent variables for optimization of BCR-NLC in BBD.

Independent Variables	Levels Used		
	−1	0	1
Solid lipid: liquid lipid ratio	70:30	80:20	90:10
Surfactant concentration (% *w*/*v*)	1.5	2.0	2.5
Stirring speed (rpm)	5000	7500	10,000
**Dependent variables**			
PS (nm) (V1)	Minimize		
PDI (V2)	Maximum		
EE (%) (V3)	Maximum		

## Data Availability

Data are contained within the article and Supplementary Materials.

## Supplementary Materials

The following supporting information can be downloaded at: https://www.mdpi.com/article/10.3390/ph17060720/s1, Table S1. Effect of type of surfactant. Table S2. Accuracy of the proposed bioanalytical HPLC method. Table S3. Precision study of the proposed bioanalytical HPLC method. Table S4. LOD and LOQ. Figure S1. Chromatogram of BCR. Figure S2. Calibration curve of BCR in plasma. Figure S3. Calibration curve of BCR in Brain Homogenate.
