# Supplementary material for: Box-Behnken Design-Based Optimization and Evaluation of Lipid-Based Nano Drug Delivery System for Brain Targeting of Bromocriptine
Source: Pharmaceuticals (Basel). 2024 Jun 2;17(6):720. doi: 10.3390/ph17060720 (PMC11206536; doi:10.3390/ph17060720)
Supplement: Supplementary file 1 [file pharmaceuticals-17-00720-s001.zip › pharmaceuticals-2982925-supplementary.pdf]

pharmaceuticals

MDPI

Supplementary Data

Article

Box-Behnken Design-Based Optimization and Evaluation of Lipid-Based Nano Drug Delivery System for Brain Targeting of Bromocriptine

Supplementary Data

Effect of Different types of surfactants

Different surfactants were screened for nanoparticle PS and PDI, such as Tween 20, Tween 80, Cremophore EL, and Pluronic F68 (Table S1). Among different surfactants, the smallest PS (274.8±4.5) was obtained with Tween 80 with low PDI (0.288±0.0025). Thus Tween 80 was chosen for the preparation of SLN.

Supplementary Table S1. Effect of type of surfactant

Surfactant	PS (nm)	PDI
**Tween 20**	294.9±12.5	0.346±0.0031
**Tween 80**	274.8±4.5	0.288±0.0025
**Cremophor EL**	1780.2±18.5	0.887±0.0085
**Pluronic F68**	354±15.7	0.324±0.0065

Analytical method development and validation of BCR using RP HPLC

Different mobile phases and flow rates were studied to select the mobile phase, which gives the best peak. The best peak was obtained with ACN: ammonium acetate (70:30), hence selected as a Mobile phase. The flow rate was set at 1mL/min. The analyte peak obtained with this condition was well-defined and free from tailing and the retention time was found to be 3.6 min (Figure S1).

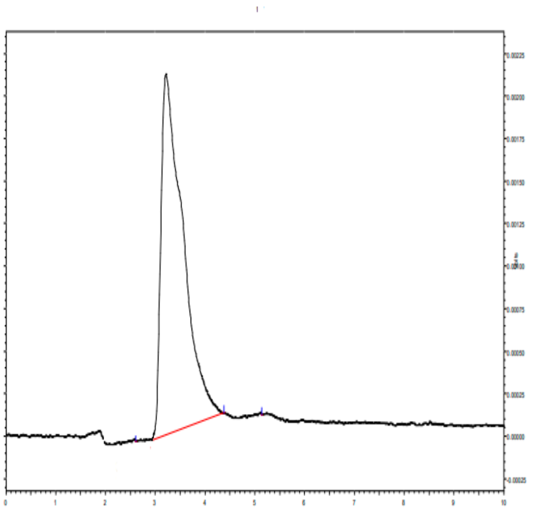

Supplementary Figure S1. Chromatogram of BCR

i. Specificity:

The proposed method provides good separation, with no extra peaks detected near the drug retention time due to endogenous components.

ii. Linearity and calibration curves:

The calibration graph of peak ratio versus concentration for plasma and brain was plotted is shown in figure S2 and figure S3 respectively. The linearity was observed in the range from 2-10 ng/ml.

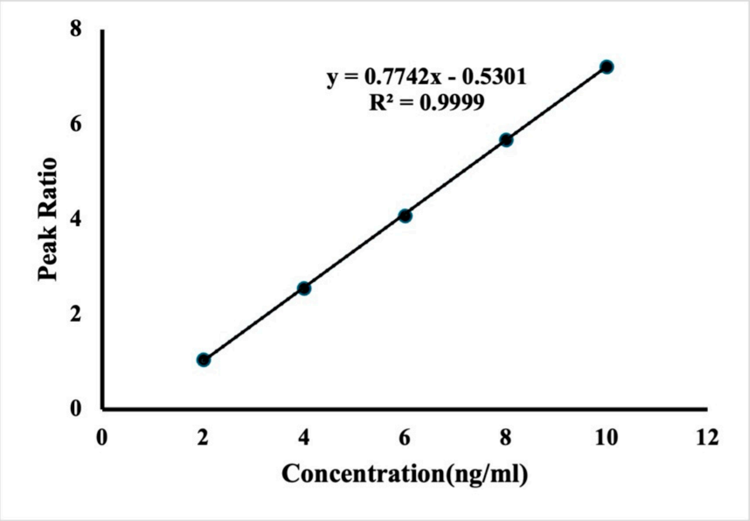

Supplementary Figure S2. Calibration curve of BCR in plasma

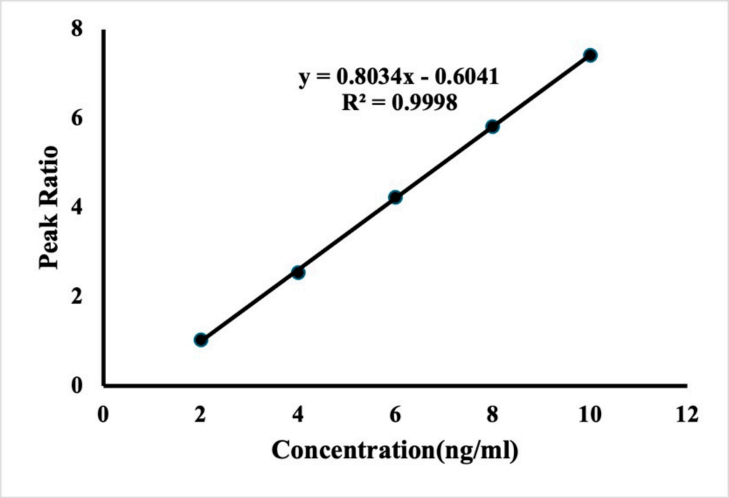

Supplementary Figure S3. Calibration curve of BCR in Brain Homogenate

iii. Accuracy

Supplementary Table S2. Accuracy of the proposed bioanalytical HPLC method

conc. (ng/mL)	Recoverd conc. (ng/mL)		Recovery (%)
	Mean±S.D.	R.S.D (%)	
**4**	3.85±0.04	1.04	96.25
**6**	5.84 ±0.06	1.12	97.3
**8**	7.7±0.1	1.35	96.25

The mean extraction recoveries of BCR at different concentrations (Low, Medium, High) was found to

be >95% (Table S2). This indicates that the present method was highly accurate and acceptable for the intended use.

iv. Precision

A precision study had been performed by measuring the RSD of different concentrations to check the interday and intraday variation. Results showed < 5% of RSD values, which is acceptable (Table S3).

Supplementary Table S3. Precision study of the proposed bioanalytical HPLC method

	**Intraday Precision**		**Interday Precision**	
**conc. (ng/mL)**	Recoverd conc. (ng/mL)		Recoverd conc. (ng/mL)	
	Mean±S.D.	R.S.D (%)	Mean±S.D.	R.S.D (%)
**4**	3.84±0.05	1.4	3.78±0.08	2.35
**6**	5.81 ±0.07	1.2	5.737±0.14	2.53
**8**	7.7±0.1	1.30	7.67±0.19	2.55

v. LOD and LOQ

LOD was determined at a signal-to-noise ratio of ≥ 3 whereas LOQ was determined at a signal-to-noise ratio of ≥ 10 (Table S4).

Supplementary Table S4. LOD and LOQ

	**Plasma**	**Brain**
**LOD**	0.12 ng/mL	0.15 ng/mL
**LOQ**	0.40 ng/mL	0.51 ng/mL
